# Gene set enrichment analysis of the bronchial epithelium implicates contribution of cell cycle and tissue repair processes in equine asthma

**DOI:** 10.1038/s41598-018-34636-9

**Published:** 2018-11-06

**Authors:** Laurence Tessier, Olivier Côté, Mary Ellen Clark, Laurent Viel, Andrés Diaz-Méndez, Simon Anders, Dorothee Bienzle

**Affiliations:** 1 Department of Pathobiology, University of Guelph, 50 Stone Road East, Guelph, Ontario, N1G 2W1 Canada; 2 Department of Clinical Studies, University of Guelph, 50 Stone Road East, Guelph, Ontario, N1G 2W1 Canada; 3 Zentrum für Molekulare Biologie der Universität Heidelberg (ZMBH), Im Neuenheimer Feld 282, 69120 Heidelberg, Germany; 4 Present Address: BenchSci, 559 College St, Toronto, ON M6G 1A9 Canada; 5 Present Address: BioAssay Works LLC, 10075 Tyler Place, Suite 18, Ijamsville, MD 21754 USA; 6 Present Address: Asia Pacific Centre for Animal Health (APCAH), Faculty of Veterinary and Agricultural Sciences, The University of Melbourne, Victoria, 3010 Australia

## Abstract

Severe equine asthma is a chronic inflammatory condition of the lower airways similar to adult-onset asthma in humans. Exacerbations are characterized by bronchial and bronchiolar neutrophilic inflammation, mucus hypersecretion and airway constriction. In this study we analyzed the gene expression response of the bronchial epithelium within groups of asthmatic and non-asthmatic animals following exposure to a dusty hay challenge. After challenge we identified 2341 and 120 differentially expressed genes in asthmatic and non-asthmatic horses, respectively. Gene set enrichment analysis of changes in gene expression after challenge identified 587 and 171 significantly enriched gene sets in asthmatic and non-asthmatic horses, respectively. Gene sets in asthmatic animals pertained, but were not limited, to cell cycle, neutrophil migration and chemotaxis, wound healing, hemostasis, coagulation, regulation of body fluid levels, and the hedgehog pathway. Furthermore, transcription factor target enrichment analysis in the asthmatic group showed that transcription factor motifs with the highest enrichment scores for up-regulated genes belonged to the E2F transcription factor family. It is postulated that engagement of hedgehog and E2F pathways in asthmatic horses promotes dysregulated cell proliferation and abnormal epithelial repair. These fundamental lesions may prevent re-establishment of homeostasis and perpetuate inflammation.

## Introduction

Severe equine asthma (recurrent airway obstruction, heaves) is a naturally occurring lung condition affecting horses that are chronically exposed to airborne environmental dust and microbial components^1^. Features of exacerbated disease include excessive mucus production, cough, neutrophilic airway inflammation, bronchial hyperreactivity, and bronchospasm. Chronic inflammation in the lower airway of affected horses leads to epithelial hyperplasia, smooth muscle hyperplasia and hypertrophy, and fibrosis, culminating in irreversible airway remodeling^2–5^.

The specific pathways underlying the condition remain poorly understood. Genetic predisposition and environmental triggers are thought to be the major factors leading to the development of disease^6^. Inheritance patterns are complex, implying genetic heterogeneity^7^ and suggesting mechanistic variation across different kinships. In humans, childhood asthma associated with atopy was strongly linked with a Th2 response^8^, which prompted investigation of Th2 pathways in severe equine asthma. However, human adult-onset asthma, which resembles severe equine asthma, has not consistently been linked to a particular Th1 or Th2 response^9^. Data regarding a particular dominant Th set have also been equivocal in horses, and a mixed immune response with biological complexity greater than that of the Th1/Th2 paradigm is considered likely^10–14^. Other factors such as the heterogeneity of cells and tissues assessed, degree of asthmatic exacerbation in subjects, frequency and timing of samples in a dynamic response, and the limited number of pathway-specific markers, are apt to contribute to diverse conclusions.

Transcription factors assessed to date in the equine asthmatic inflammatory response include activator protein-1 (AP-1), cyclic AMP response element binding protein (CREB), CAAT/enhancer binding protein (C/EBP), GATA-3 and nuclear factor (NF)-κB^15–17^. Activity of AP-1 in bronchial brushing (BB) cells and NF-κB in bronchoalveolar lavage (BAL) cells positively correlated with active disease, while CREB activity was higher in BB cells of asthmatic than control horses two months after challenge^15–17^. Neither differences in target binding of GATA-3 and C/EBP nor specific targets of transcription factors were identified. Target enrichment analysis in peripheral blood mononuclear cells (PBMC) from asthmatic horses also suggested hypoxia-inducible factor 1 (HIF-1) as a potential regulator^18^. Serum response factor (SRF) and its co-factor myocardin (MYOCD) were increased in airway smooth muscle (ASM) cells of peripheral but not central airways of asthmatic horses, and were considered to contribute to ASM hypertrophy^19^.

The bronchial epithelium has a major role in the development of asthma. In human asthma, the airway epithelial barrier function is physically and functionally impaired, which manifests with disrupted tight junctions, altered innate immune products and compromised regeneration of differentiated cell types^20,21^. SCGB1A1 production by the bronchial epithelium is one such specialized epithelial function lost in asthmatic horses, and indicates absence of mature club (Clara) cells, which in turn implies limited anti-inflammatory functions of airway secretions^22,23^. Activity of p65 NF-κB homodimer in BAL leukocytes, and production of certain epithelial cytokines, was altered in asthmatic horses^16,24^, but changes in the airway epithelium have not been comprehensively analyzed.

The evolution of RNA-sequencing (RNA-seq) technology has enabled large-scale analysis of gene expression, a valuable tool for quantitative and unbiased assessment. In turn, gene set enrichment analysis aims to determine whether defined sets of related or interconnected genes identified by RNA-seq significantly differ between phenotypes. The relationship between statistically significant and biologically significant RNA-seq gene expression changes remains to be fully explored in most experimental systems, and may vary across different genes. Therefore, stringent statistical cutoffs applied to genome-wide sequencing data may impair the ability to detect biologically meaningful changes. Gene set enrichment performed with software such as Gene Set Enrichment Analysis (GSEA) leverages the unbiased nature of RNA-seq data by by-passing pre-determined statistical cut-offs at the single gene level in order to reveal cellular processes associated with a particular phenotype^25^.

Previous work identified differences in gene expression between asthmatic and non-asthmatic horses following challenge. In the current study, using the same initial data set, we investigated changes in gene expression associated with exacerbated equine severe asthma within groups of horses. RNA-seq data from the asthmatic and the non-asthmatic group were analyzed for differential expression before and after challenge, and results were then assessed for gene set enrichment.

## Results

### Differential expression analysis

**Figure 1:**
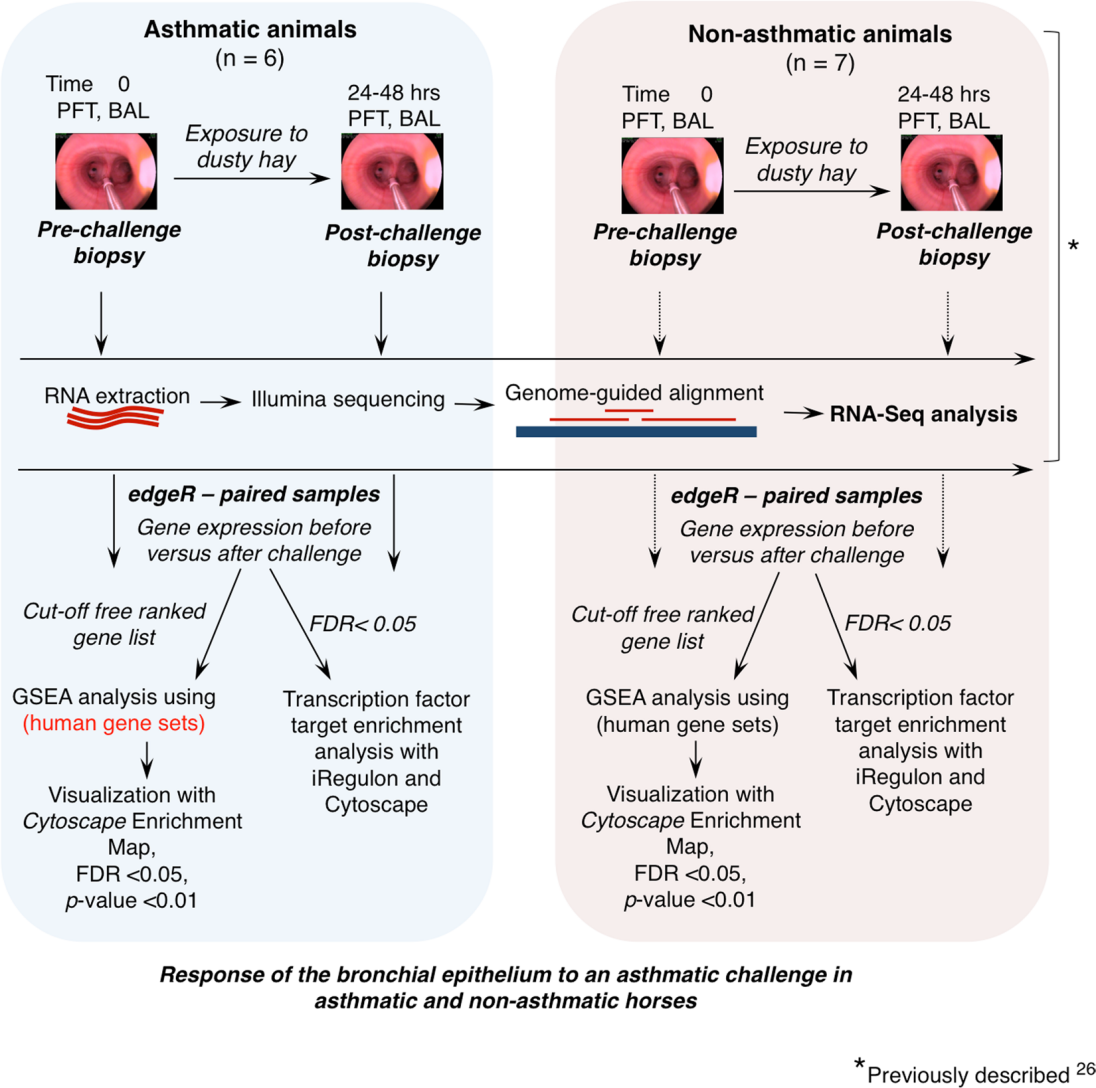
Outline of study design and analysis.

**Figure 2:**
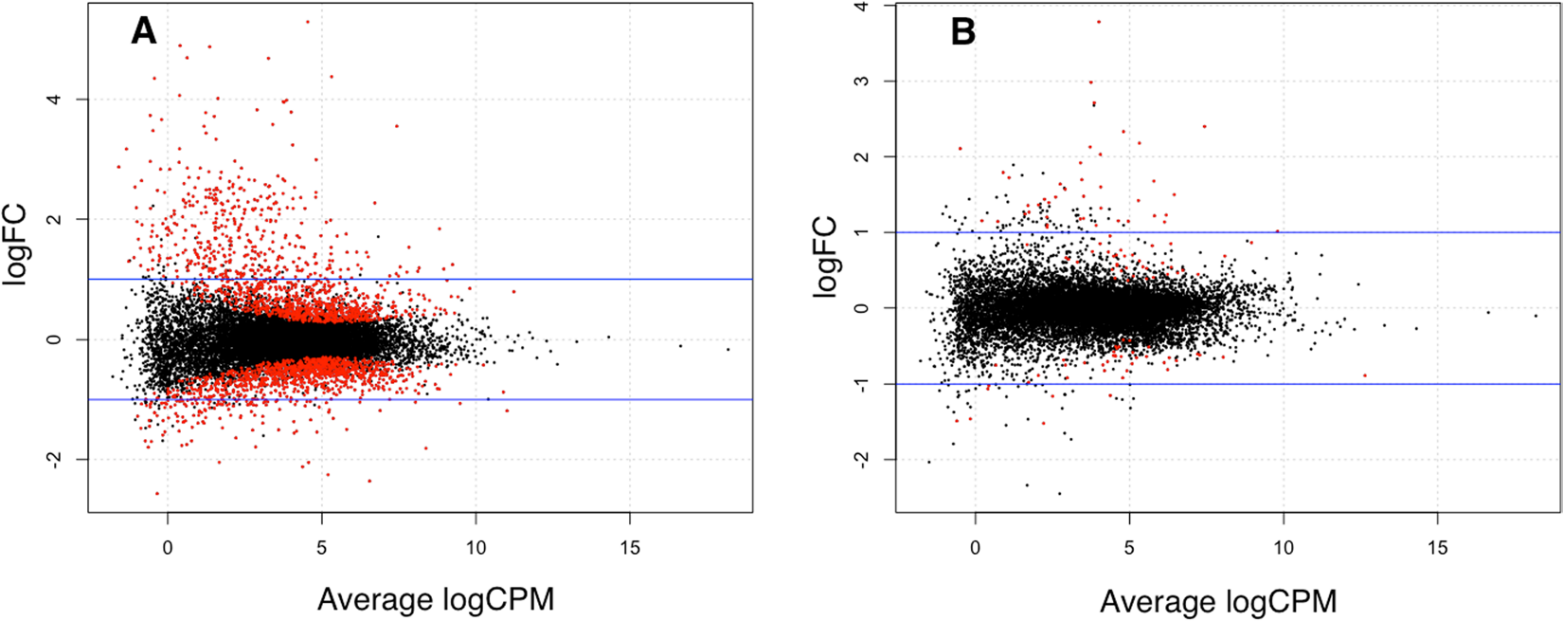
EdgeR smear plot showing the log fold-change (FC, y-axis) versus the average log count per million (CPM, x-axis) for the change in gene expression due to asthmatic challenge in horses with asthma (**A**), and in horses without asthma (**B**). Red dots represent the genes that differ significantly in horses with asthma before and after challenge (**A**), and in horses without asthma before and after challenge (**B**). Horizontal blue lines delineate 1-fold change. Significance set at FDR < 0.05. Differentially expressed genes can be observed in both groups, but a greater number were observed in horses with asthma.

Differential expression analysis of the bronchial epithelium transcriptome following challenge (Fig. 1) within groups of horses with (Fig. 2A, Suppl. Table 1) and without (Fig. 2B, Suppl. Table 2) asthma yielded 2341 and 120 differentially expressed (DE) genes, respectively.

### Gene set enrichment analysis

**Figure 3:**
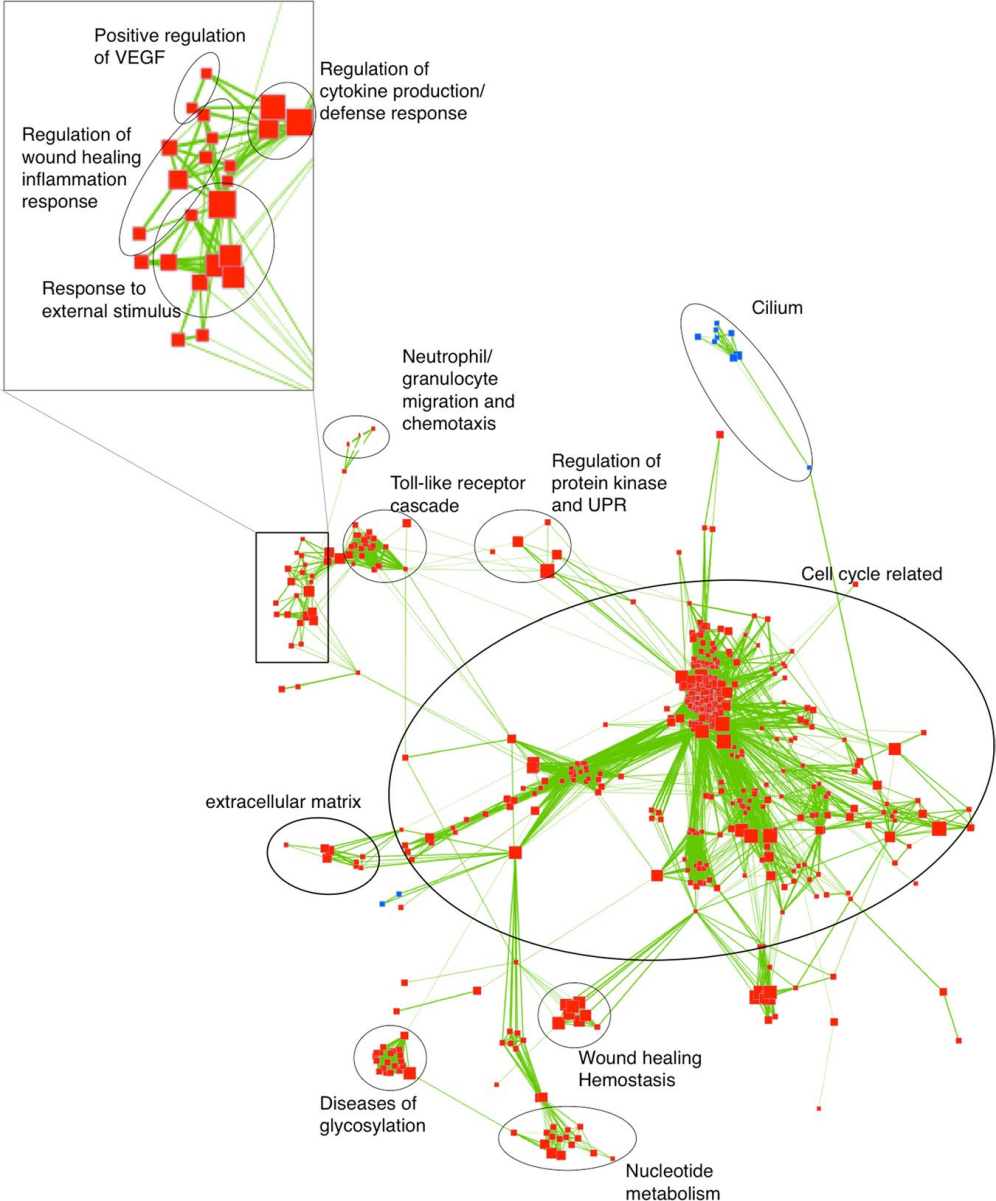
Results of gene set enrichment analysis (GSEA) of horses with asthma visualized with Cytoscape Enrichment Map. Each node (square) corresponds to a gene set either up-regulated (red) or down-regulated (blue) in response to asthmatic challenge. Edges (green lines) link sets with shared genes, and thickness of lines correlates with the number of genes in common between two sets. Only gene sets with FDR < 0.05 and *p* < 0.01 were included in visualizations; disconnected nodes and small clusters were removed. Clusters of gene sets involved in cell cycle, Toll-like receptor (TLR) pathways, wound healing, glycosylation and other inflammatory and defense responses are outlined in black circles.

**Figure 4:**
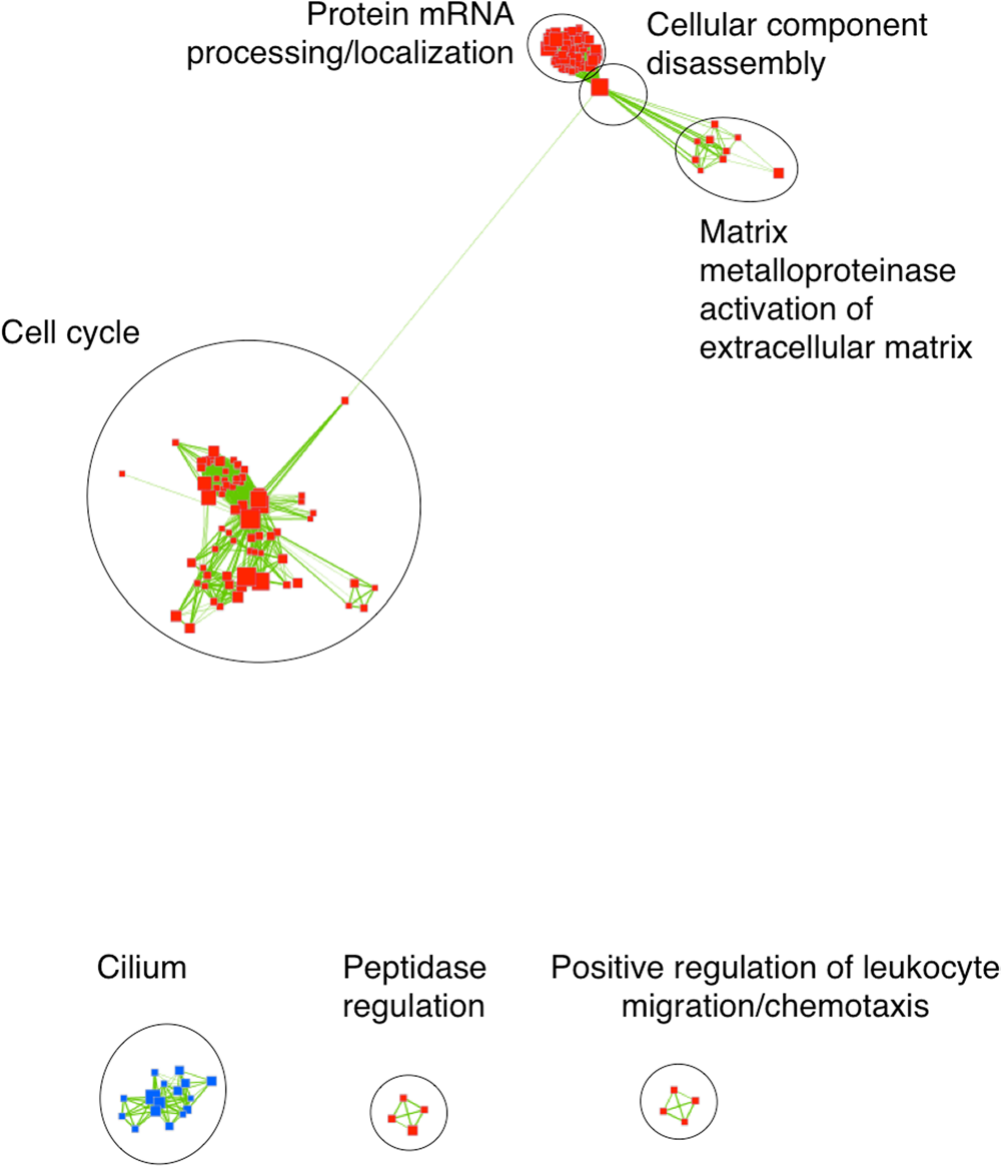
Results of gene set enrichment analysis (GSEA) of horses without asthma visualized with Cytoscape Enrichment Map; labels as in Fig. 3. Only gene sets with FDR < 0.05 and *p* < 0.01 were included in visualizations. Disconnected nodes and small clusters were removed. Clusters of gene sets involved in cell cycle, mRNA processing, cell metabolism and ECM are apparent (black circles), and small aggregates of gene sets with function in leukocyte chemotaxis, peptidase and cilium.

Within the asthmatic group, 587 gene sets were significantly enriched and linked by 18,777 edges while in non-asthmatic horses 171 gene sets were significantly enriched and linked by 2326 edges. Results of GSEA (FDR < 0.05, *p* < 0.001) were visualized using the Enrichment Map plugin available for Cytoscape (Figs 3 and 4, detailed results in Supp. Tables 3–6). In Fig. 3, linked gene sets present only in horses with asthma are shown. Gene sets involved in cell cycle regulation dominated, and genes involved in mechanisms such as inflammation/immune-response, metabolism, extracellular matrix (ECM) degradation, and protein translation and processing (Fig. 3; Suppl. Tables 3–4) were also enriched. Such gene sets showed high redundancy as indicated by tight clusters of nodes (Figs 3 and 4, red and blue squares), in particular for cell cycle, Toll-like receptor (TLR) pathway, wound healing, glycosylation and other inflammatory and defense response gene sets. In horses without asthma, similar but fewer enriched gene sets were noted (Fig. 4; Suppl. Tables 5–6). Enriched gene sets suggest concurrent expression of multiple genes in related pathways.

### Enrichment of neutrophil migration and chemotaxis gene sets

**Figure 5:**
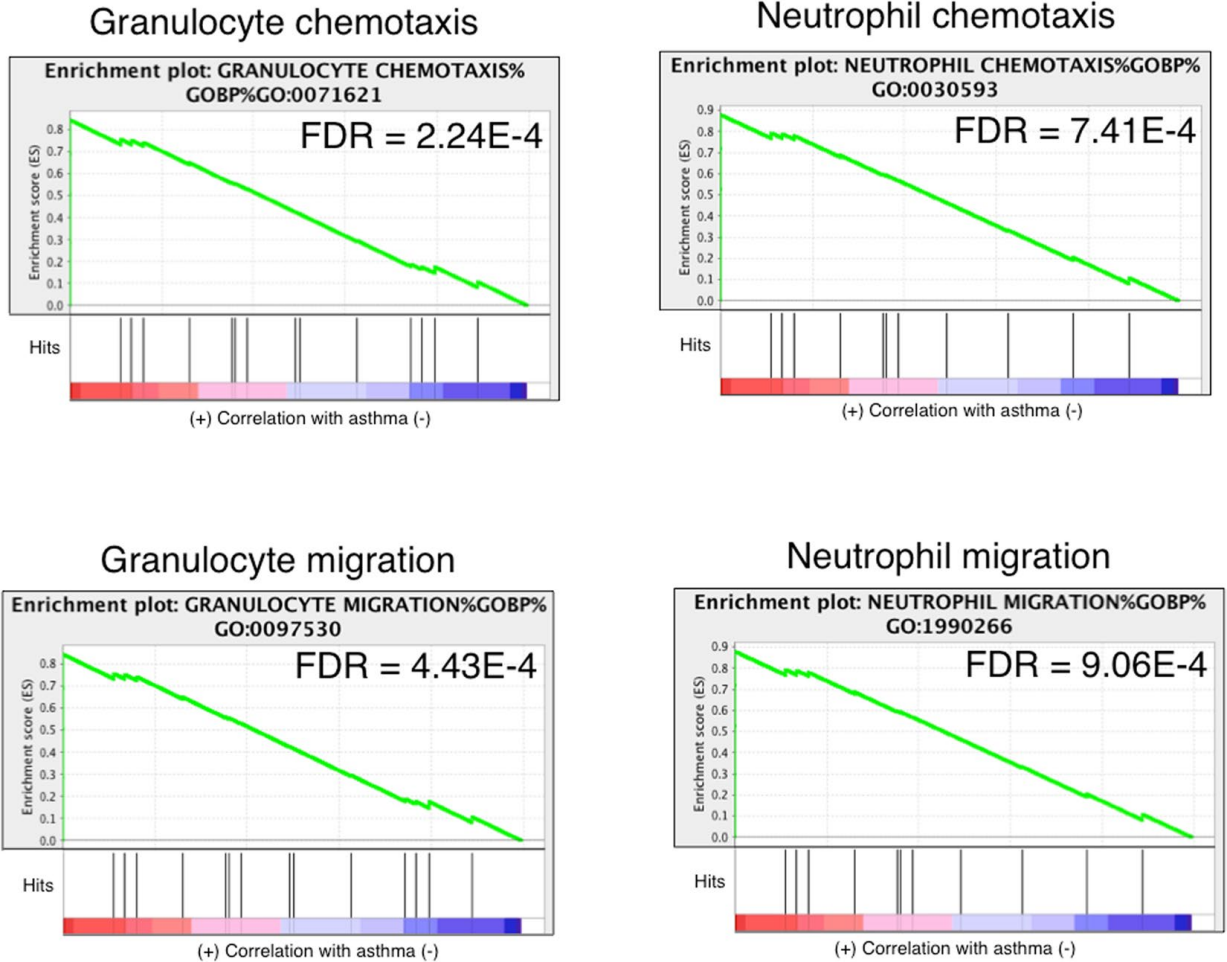
In horses with asthma, gene sets for leukocyte chemotaxis and migration were significantly enriched. The y-axis represents enrichment score (ES) and on the x-axis are genes (vertical black lines) represented in gene sets. The green line connects points of ES and genes. ES is the maximum deviation from zero as calculated for each gene going down the ranked list, and represents the degree of over-representation of a gene set at the top or the bottom of the ranked gene list. The colored band at the bottom represents the degree of correlation of genes with the asthma phenotype (red for positive and blue for negative correlation). Significance threshold set at FDR < 0.05. Gene sets for leukocytes were significant only in horses with asthma.

Within the asthmatic group, significantly up-regulated gene sets with lowest rank at max metric, suggesting higher likelihood of involvement in disease process, pertained to granulocyte and neutrophil chemotaxis and migration (Fig. 5, Table 1). Additional significant gene-sets were related to phases and components of the cell cycle (Table 2). In horses without asthma, gene sets with lowest rank at max included those connected to mitosis, cytoskeleton, protein binding and leukocyte migration and chemotaxis (Table 3). Gene sets linked to M phase and protein translation were identified as most highly significant (Table 4).

**Table 1:** Top up-regulated gene-sets in horses with asthma following challenge, ranked according to lowest rank at maximum score.

Name of gene set	Size of gene set	NES^^^	FDR*	Rank at max
Granulocyte chemotaxis - gobp - go:0071621	19	2.01	2.24E-04	16
Granulocyte migration - gobp - go:0097530	19	1.98	4.43E-04	16
Neutrophil chemotaxis - gobp - go:0030593	15	1.94	7.41E-04	16
Neutrophil migration - gobp - go:1990266	15	1.93	9.06E-04	16
Myeloid leukocyte migration - gobp - go:0097529	31	1.80	5.83E-03	16
Leukocyte chemotaxis - gobp - go:0030595	46	1.69	2.03E-02	16
Positive regulation of cytokine secretion - gobp - go:0050715	33	1.61	4.11E-02	68
Negative regulation of inflammatory response - gobp - go:0050728	30	1.67	2.56E-02	134
Cell separation after cytokinesis - gobp - go:0000920	18	1.73	1.35E-02	140
Chemokine receptor binding - gomf - go:0042379	22	1.95	5.97E-04	164

^^^Normalized enrichment score.

^*^False discovery rate.

**Table 2:** Top up-regulated gene-sets in horses with asthma following challenge, ranked according to FDR.

Name of gene set	Size of gene set	NES^^^	FDR*	Rank at max
M phase - reactome - react_910.4	213	2.68	<0.001^a^	1633
Cell cycle, mitotic - reactome - react_152.7	373	2.67	<0.001^a^	1633
Mitotic metaphase and anaphase - reactome - react_150314.2	150	2.64	<0.001^a^	1624
Cell cycle - reactome - react_115566.4	430	2.63	<0.001^a^	1633
Mitotic nuclear division - gobp - go:0007067	126	2.61	<0.001^a^	1016
Mitotic anaphase - reactome - react_1275.3	149	2.60	<0.001^a^	1624
Nuclear division - gobp - go:0000280	168	2.59	<0.001^a^	1016
Separation of sister chromatids - reactome - react_150471.2	141	2.58	<0.001^a^	1624
Mitotic prometaphase - reactome - react_682.3	96	2.54	<0.001^a^	1515
Chromosome segregation - gobp - go:0007059	129	2.54	<0.001^a^	1186

^^^Normalized enrichment score.

*False discovery rate.

^a^Exact FDR value not detected (<1/maximum number of permutations).

**Table 3:** Top up-regulated gene-sets in non-asthmatic horses following challenge, ranked according to lowest rank at maximum score.

Name of gene set	Size of gene set	NES^^^	FDR^*^	Rank at max
Mitotic cytokinesis - gobp - go:0000281	25	1.99	2.91E-04	286
Negative regulation of protein binding - gobp - go:0032091	34	2.005	2.39E-04	288
Cytoskeleton-dependent cytokinesis - gobp - go:0061640	30	1.96	8.31E-04	288
Negative regulation of binding - gobp - go:0051100	58	1.87	7.87E-03	288
Aurora a signaling - pathway interaction database nci-nature curated data - aurora a signaling	25	1.84	1.36E-02	288
Regulation of protein binding - gobp - go:0043393	71	1.77	3.87E-02	288
Condensed nuclear chromosome - gocc - go:0000794	29	1.75	4.49E-02	288
Microtubule cytoskeleton organization involved in mitosis - gobp - go:1902850	24	1.75	4.35E-02	291
Condensed chromosome, centromeric region - gocc - go:0000779	26	1.94	1.46E-03	319
Regulation of leukocyte chemotaxis - gobp - go:0002688	48	1.82	1.98E-02	347

^^^Normalized enrichment score.

^*^False discovery rate.

**Table 4:** Top up-regulated gene-sets in non-asthmatic horses following challenge, ranked according to FDR.

Name of gene set	Size of gene set	NES^^^	FDR^*^	Rank at max
Translation - reactome - react_1014.4	118	2.20	<0.001^a^	1771
Cap-dependent translation initiation - reactome - react_2099.1	88	2.16	<0.001^a^	1771
3′ -utr-mediated translational regulation - reactome - react_1762.2	80	2.15	<0.001^a^	1503
M phase - reactome - react_910.4	213	2.15	<0.001^a^	1647
l13a-mediated translational silencing of ceruloplasmin expression - reactome - react_79.2	80	2.15	<0.001^a^	1503
Eukaryotic translation initiation - reactome - react_2159.5	88	2.14	<0.001^a^	1771
Protein targeting to er - gobp - go:0045047	87	2.14	<0.001^a^	1869
Mitotic prometaphase - reactome - react_682.3	96	2.13	<0.001^a^	1249
Gtp hydrolysis and joining of the 60 s ribosomal subunit - reactome - react_2085.2	81	2.13	<0.001^a^	1771
Cotranslational protein targeting to membrane - gobp - go:0006613	85	2.11	<0.001^a^	1869

^^^Normalized enrichment score.

*False discovery rate.

^a^Exact FDR value not detected (<1/maximum number of permutations).

### Enrichment of cell cycle, hedgehog and hemostasis-related gene sets

**Figure 6:**
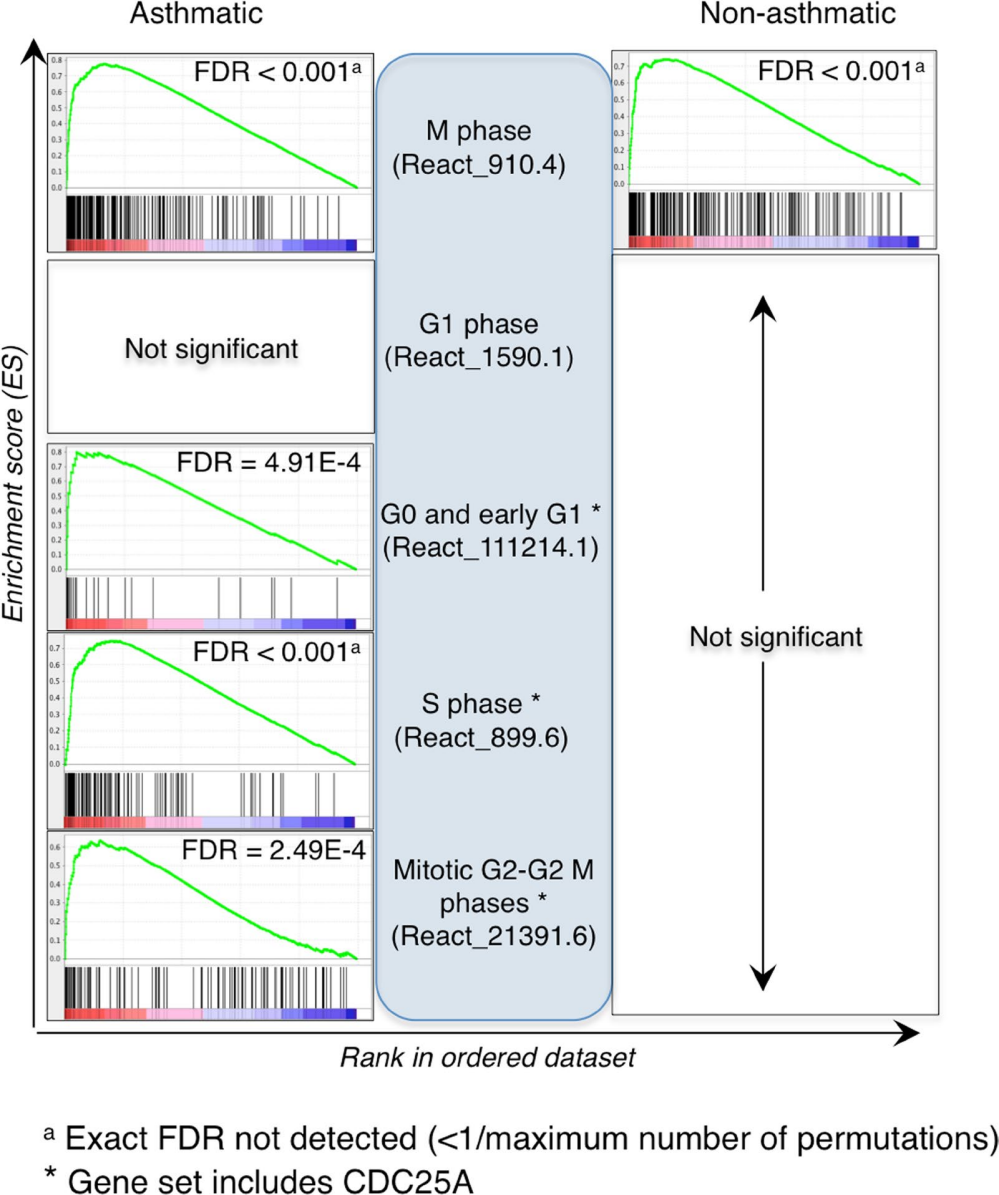
In horses with asthma, gene sets for M, G0 and early G1, S and G2-M phases of the cell cycle were significantly enriched, while in horses without asthma only gene sets for M phase of the cell cycle were significantly enriched. See caption of Fig. 5 for details.

Detailed analysis of cell cycle phases identified significantly enriched gene sets involved in all phases except G1 within the asthmatic group following challenge, while only those in M phase were significantly enriched in the non-asthmatic group (Fig. 6). High enrichment of cell cycle gene sets in the asthmatic group correlated with significantly up-regulated expression of M-phase inducer phosphatase 1 (*CDC25A*) in horses with asthma (Suppl. Table 1). Forkhead box protein M1 (*FOXM1*) was significantly up-regulated only in horses with asthma (Suppl. Table 1). Expression of *CDC25A* and *FOXM1* corresponded to enrichment in cell cycle gene sets (Fig. 3).

**Figure 7:**
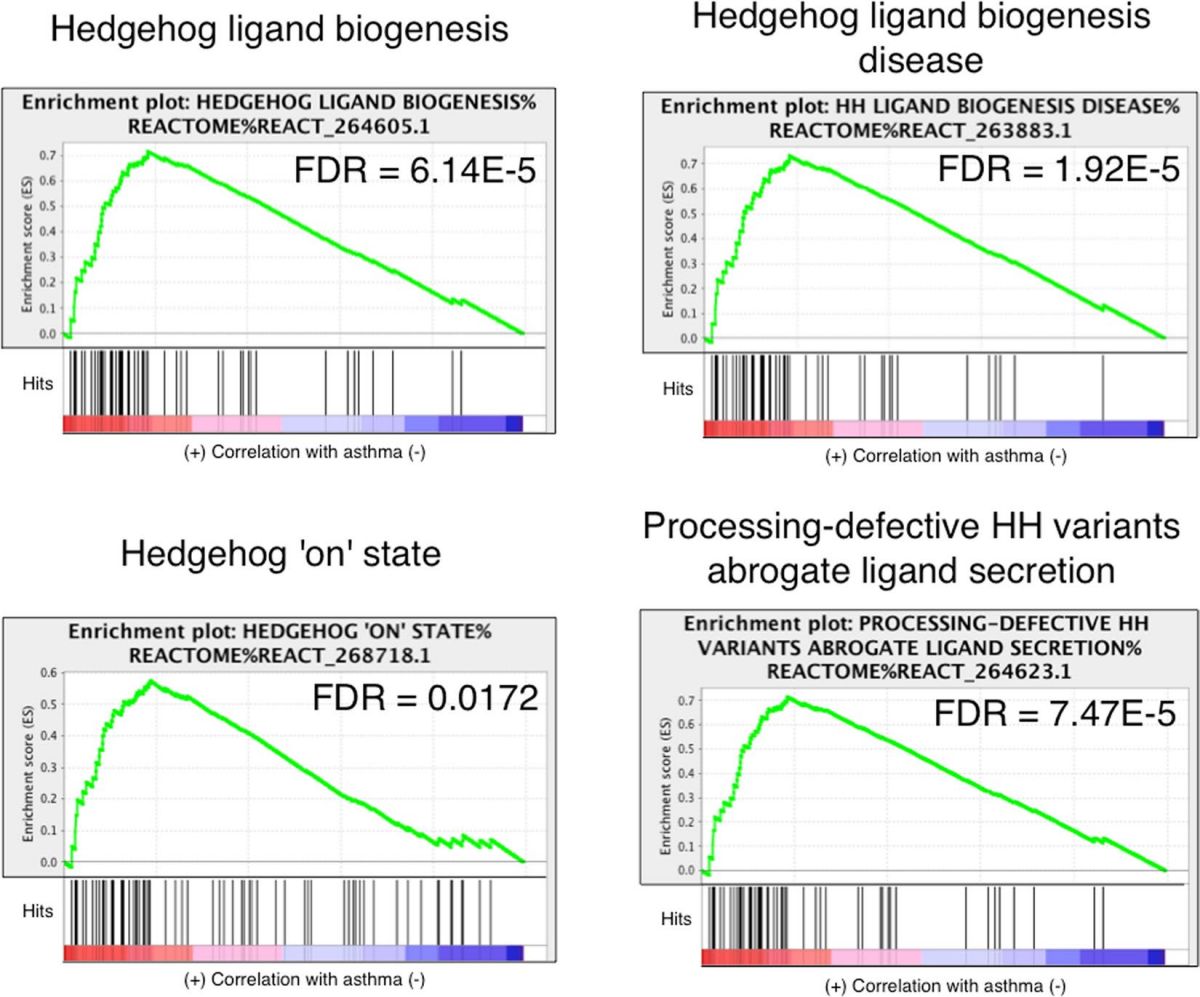
Curves of GSEA enrichment scores for Hedgehog-associated pathways in horses with asthma. Gene sets significantly enriched only in horses with asthma suggests differential activity of the pathway in the two groups. See caption of Fig. 5 for details.

**Figure 8:**
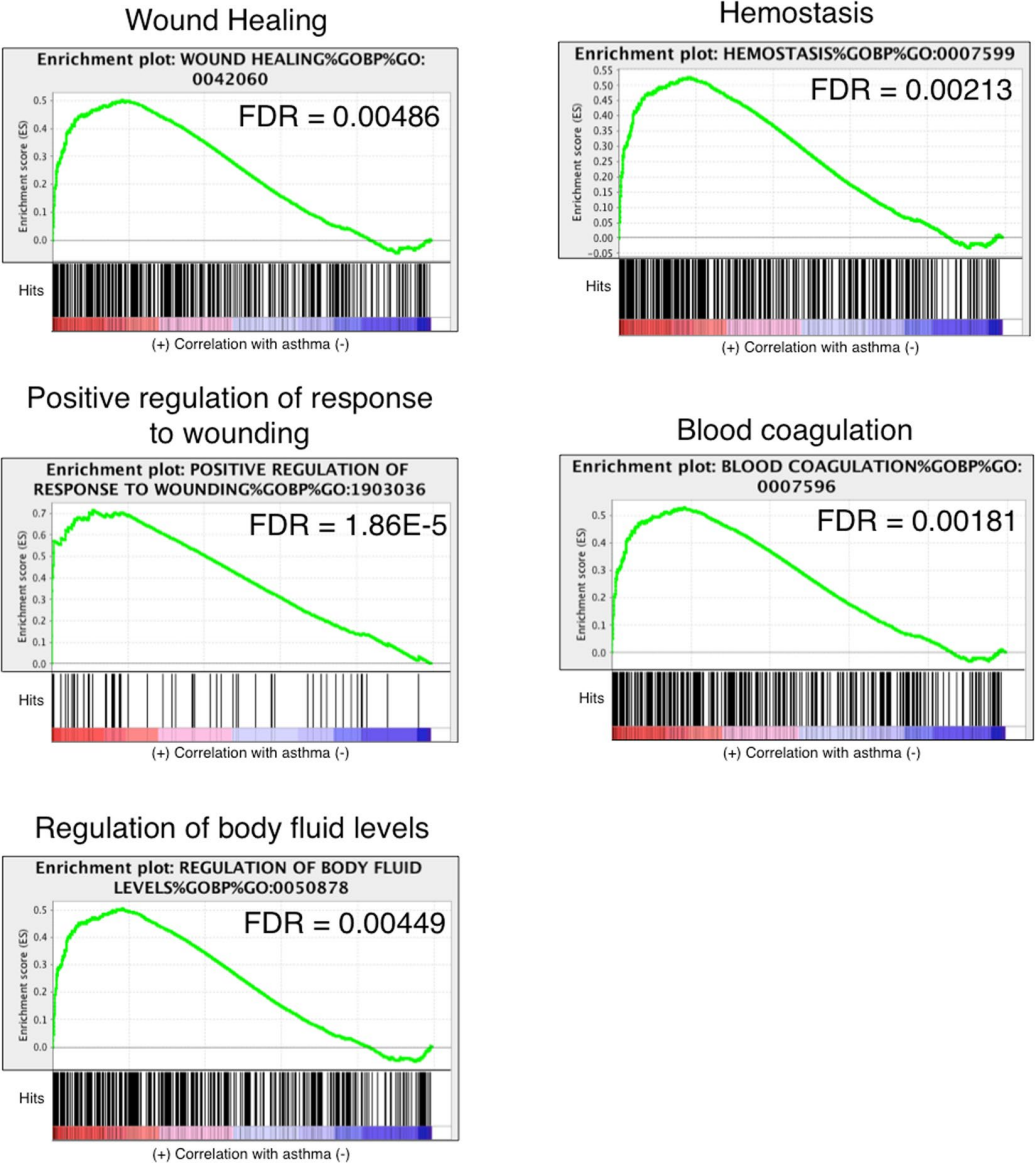
Curves of GSEA enrichment scores for wound healing and hemostasis-associated pathways in horses with asthma. These gene sets were significantly enriched only in horses with asthma, suggesting higher activity of pathways related to these processes. See caption of Fig. 5 for details.

Multiple gene sets interacting with hedgehog (Hh) were identified as significantly enriched within the asthmatic but not the non-asthmatic group (Fig. 7): “hedgehog ‘on’ state” (FDR = 0.0172; REACT_268718.1), “hedgehog ligand biogenesis” (FDR = 6.14E^−05^; REACT_264605.1), “Hh ligand biogenesis disease” (FDR = 1.92E^−05^; REACT_263883.1) and “processing-defective hh variants abrogate ligand secretion” (fdr = 7.47e^−05^; react_264623.1). Two gene sets linked to promotion of Wnt signaling were also significantly enriched in only asthmatic horses: “Deletions in the axin genes in hepatocellular carcinoma result in elevated wnt signalling” (FDR = 2.10E^−03^; REACT_264286.1) and “Axin mutants destabilize the destruction complex, activating Wnt signaling” (FDR = 2.57E^−03^; REACT_264496.1) (Suppl. Table 3). Significant up-regulation of hypoxia-inducible factor 1α (*HIF1α*) and enrichment of the p53-hypoxia pathway (MSIGDB_C2) gene set was detected only in horses with asthma. Similarly, gene sets involved in “response to wounding and hemostasis” (Fig. 8), were significant only in horses with asthma. Complete lists of gene sets are in Suppl. Tables 3–6.

### iRegulon

Within the asthmatic group, a large number of significantly up-regulated genes were identified as potential targets of E2F transcription factors. The ten transcription factor motifs with the highest enrichment score in asthmatic and non-asthmatic horses are listed in Tables 5 and 6, respectively. Of note, E2F2, E2F3 and E2F8 were significantly up-regulated in asthmatic horses while only E2F1 was significantly down-regulated in non-asthmatic horses. Complete lists of targets for each of the transcription factor motifs are in Suppl. Tables 7–10.

**Table 5:** Most enriched transcription factor motifs among significantly up- and down-regulated genes in horses with asthma following challenge.

Transcription factor motif ID	NES^^^	Transcription factors
***Up-regulated genes***		
Transfac_pro-M00920	8.72	E2F7, TFDP1, E2F3*, E2F4, E2F1, TFDP3, E2F5, E2F2*
Transfac_pro-M00919	8.72	E2F1, TFDP1, E2F3*, E2F4, TFDP3, E2F7, E2F2*, E2F5
Swissregulon-E2F1.5.p2	8.63	E2F2*, E2F4, E2F1, E2F5, E2F3*, TFDP1, E2F7, TFDP3
Homer-M00032	8.56	E2F7, E2F4, E2F6, E2F1
Transfac_pro-M00740	8.30	E2F1, E2F3*, E2F4, E2F5, E2F2*, TFDP1, E2F7, TFDP3
Transfac_pro-M00738	8.29	E2F4, E2F1, E2F5, E2F3*, E2F2*, TFDP1, E2F7, TFDP3
Jaspar-PF0069.1	8.25	E2F4, E2F7, E2F6, E2F1, E2F8*
Homer-M00028	8.18	E2F1, E2F4, E2F3*, E2F7, TFDP1, E2F5, E2F2*, TFDP3
Transfac_pro-M00939	8.15	E2F1, TFDP1, E2F4, E2F3*, TFDP3, E2F7, E2F5, E2F2*
Transfac_public-M00050	8.11	E2F4, TFDP1, E2F3*, E2F1, TFDP3, E2F7, E2F5, E2F2*
***Down-regulated genes***		
Transfac_pro-M02790	5.24	RFX7, **RFX5**^#^
Transfac_pro-M02789	4.69	RFX4, **RFX5**^#^, RFX7
Taipale-SGTTGCYARGCAACS-RFX4-DBD	4.58	RFX4, RFX3, **RFX2**^#^, **RFX1**^#^
Taipale-SGTTGCYARGCAACS-Rfx2-DBD	4.57	RFX3, **RFX2**^#^, RFX4, **RFX1**^#^
Taipale-GTAAACAW-FOXO3-full	4.57	FOXO3, FOXC2, FOXO1, FOXF2, FOXD1, FOXD2, HLTF, **FOXO4**^#^, FOXO6, FOXA1, FOXA2, FOXJ2, FOXI1, FOXK1
Swissregulon-FOX_D1-D2_.p2	4.47	FOXD1, FOXD2, FOXO1, HLTF, FOXC2, FOXF2, FOXO3, FOXA2, FOXA1, FOXO6, FOXJ2, **FOXO4**^#^, FOXK1
Taipale-SGTTGCYARGCAACS-RFX2-DBD	4.26	**RFX2**^#^, RFX3, RFX4
Yetfasco-501	4.26	N/A
Yetfasco-1452	3.80	HLF, **TEF**^#^, NFIL3, **DBP**^#^, ATF2
Homer-M00165	3.80	**RFX5**^#^, **RFX1**^#^, RFX7

^^^Normalized enrichment score.

*Transcription factors significantly up-regulated following challenge.

^**#**^Transcription factors significantly down-regulated following challenge.

**Table 6:** Most enriched transcription factor motifs among significantly up- and down-regulated genes in horses without asthma following challenge.

Transcription factor motif ID	NES	Transcription factors
***Up-regulated genes***		
jaspar-MA0314.1	6.01	NFYB, POLE3, NFYC, NFYA
yetfasco-1536	5.73	NFYB, POLE3, NFYA, NFYC
homer-M00123	5.58	RELA, NFKB2, REL, NFKB1, BCL3, OVOL2, STAT6, EBF1
taipale-RRGGTCAAAGTCCRNN-HNF4A-DBD	5.49	HNF4A, NR2F1, NR2F2, HNF4G, RXRG, PPARG, RXRB, RXRA, NR2C2
jaspar-MA0316.1	5.27	NFYC, POLE4, NFYA, NFYB
transfac_public-M00288	5.27	NFYB, NFYA, POLE3, NFYC
elemento-CTGGCCA	5.23	N/A
yetfasco-1537	5.19	NFYC, POLE4, NFYA, NFYB
jaspar-MA0060.1	5.09	NFYA, NFYB, NFYC, YBX1, POLE3, POLE4
transfac_public-M00287	4.91	NFYA, NFYB, NFYC, YBX1, POLE3, POLE4
***Down-regulated genes***		
Taipale-SGTTGCYARGCAACS-RFX4-DBD	5.77	RFX4, RFX3, **RFX2**^#^, RFX1
Transfac_pro-M00481	5.67	AR, PGR, NR3C1, NR3C2
Factorbook-NR3C1	5.39	NR3C1, AR, PGR, NR3C2, HSF1
Yetfasco-658	5.38	
Transfac_pro-M02789	5.17	RFX4, RFX5, RFX7
Taipale-RRGWACANNNTGTWCYY-AR-DBD	5.15	AR, NR3C1, NR3C2, PGR
Transfac_pro-M02465	4.96	HMGB4, TOX, TOX2, TOX4, TOX3, TBP, TBPL2, TBPL1
Transfac_pro-M02790	4.82	RFX7, RFX5
Transfac_pro-M01201	4.77	AR, NR3C1, PGR, NR3C2
Swissregulon-AR.p2	4.75	AR, PGR, NR3C1, NR3C2, HSF1

^^^Normalized enrichment score.

*Transcription factors significantly up-regulated following challenge.

^**#**^Transcription factors significantly down-regulated following challenge.

## Discussion

The goal of this study was to identify key pathways in the pathogenesis of asthma through identification and analysis of gene sets associated with the response to challenge in a group of asthmatic horses. This study built upon our previous work where bronchial biopsies from asthmatic and non-asthmatic horses were obtained before and after a challenge (dusty hay), and differences in gene expression between the two groups were determined^26^. In the current study, the gene expression response to an asthmatic challenge was analyzed for each group separately, and then gene sets related by biological process or regulation were identified. For this purpose, GSEA was applied to evaluate whether genes that were *a priori* assigned to a specific biological process (gene set) were associated with the asthmatic phenotype^25^.

GSEA builds upon gene expression data derived from RNA-seq to cluster genes based on common functions, locations, pathways, interactions or other connecting properties. Enrichment Map software with the Cytoscape plugin was applied to visualize GSEA output and to facilitate interpretation of results. After differential expression analysis with edgeR, data from each group of animals were further analyzed to identify significantly enriched gene sets and their linked edges. Edges reflect genes shared within a gene set, with the thickness of an edge corresponding to the number of overlapping genes, and similar gene sets being clustered together^26^. The number of genes with expression that differed significantly within groups of asthmatic and non-asthmatic horses after exposure to challenge was 2,341 and 120, respectively. Analysis yielded 587 significantly enriched gene sets linked by 18,777 edges, and 171 gene sets linked by 2,326 edges, respectively, which was in approximate relation to the number of differentially expressed genes. The enrichment map highlighted many closely positioned groups of gene sets, reflecting a high degree of redundancy of genes within sets. In addition, one or several genes linked most gene sets, which implies that most pathways and networks responsible for specific features of asthma were related.

### Neutrophil migration and chemotaxis gene sets

Neutrophil influx into airways is a hallmark of equine severe asthma^27^. Neutrophil and granulocyte migration and chemotaxis gene sets were ranked at max classification in asthmatic horses. Rank at max classification represents the maximum enrichment score (ES), which in turn is the maximal deviation from zero calculated for each gene descending the ranked list. Therefore, the rank represents the degree of over-representation of a gene set at the top or bottom of the ranked gene list. Enriched genes involved in neutrophil and granulocyte migration and chemotaxis were identified between positions 3 and 16 of the ranked list in asthmatic horses, yielding the highest score of all gene sets (Table 1). Hence, the top genes differentially expressed in the epithelium of horses after asthmatic challenge are highly enriched for neutrophil migration and chemotaxis, which is consistent with IL8 and CXCR2 previously identified as differentially expressed in asthmatic compared to non-asthmatic horses^28^.

### Cell cycle, hedgehog and hemostasis-related gene sets

Of particular interest was that gene sets affecting all phases of the cell cycle, except for part of the G1 phase, were significantly enriched in asthmatic horses while only those involved in M phase were significantly enriched in non-asthmatic horses. As apparent in Fig. 3, proximity visualization placed cell cycle gene sets very close together and linked them by a large number of edges, reflecting a high degree of redundancy. Detailed analysis of individual cell cycle phases (Fig. 6) showed that gene sets in M, G0 and early G1, and G2 were most significant in asthmatic horses. Although the significance of relative differences between groups cannot be derived from this analysis, we hypothesize that processes in G0, early G1 and G2 phase are most altered in asthmatic relative to non-asthmatic horses because release of injurious mediators from granulocytes stimulates induction of cell cycling in the epithelium. In agreement with this hypothesis, *CDC25A* (ENSECAG00000016336), an M2-inducing phosphatase, was up-regulated and differentially expressed in asthmatic compared to non-asthmatic horses^28^. Altered cell cycle regulation was previously reported when peripheral blood mononuclear cells (PBMC) from asthmatic horses were exposed to hay dust extract^18^. However, that study assessed changes in PBMC, which are comprised of monocytes and lymphocytes, while here changes in the bronchial epithelium were investigated. Induction of genes associated with cell proliferation in both studies suggests that hay dust extract has a direct effect on PBMC (which may include previously *in vivo* sensitized lymphocytes), and that bronchial inflammatory cells or inhaled hay dust also stimulate epithelial cell cycling. Significant up-regulation of *FOXM1*, a regulator of G1/S and G2/M cell cycle transition, further confirmed that there was induction and progression of the cell cycle^29,30^. FOXM1 regulates *CDC25A* gene transcription by direct promoter binding and indirectly via the E2F transcription factor^31^.

Previous work showed that patched 1 (*PTCH1*) was significantly down-regulated in horses with asthma compared to non-asthmatic horses^28^. Here, the Hh-related gene sets were enriched. Activation of Hh signaling through aberrant FOXM1 and GLI Family Zinc Finger 1 (Gli1) activation was observed in colorectal epithelial cells, and was considered essential for cell growth and proliferation^32^. Therefore, it is plausible that transient down-regulation of *PTCH1* and activation of Hh signaling lead to persistent bronchial cell proliferation, which in turn may be mediated by FOXM1 and CDC25A activation.

It has been proposed that injury of terminal bronchioles in airway inflammation leads to luminal exudation of plasma and accumulation of fibrinogen, thrombin and mucus in airways, and in turn a pro-coagulant state^33^. Horses with exacerbated asthma were considered to be in a hypercoagulable state and to have systemic inflammation during active disease, with hypercoagulability persisting during remission^34^. We detected here significantly enriched gene sets associated with wound healing, hemostasis, blood coagulation and regulation of body fluid levels in the asthmatic group following challenge, lending support to linkage and importance of these pathways in equine asthma. Smooth muscle cells of the bronchioles are hypercontractile in human asthmatics^35^, and SRF and its co-factor MYOCD are thought to contribute to airway remodeling in severe equine asthma^19^. Several other genes previously identified as differentially expressed between asthmatic and non-asthmatic horses are involved in wound healing, coagulation, hemostasis and regulation of body fluids, including thrombospondin 1 *(THBS1)*, oncostatin *(OSM)*, pleckstrin *(PLEK)* and others^28^. Therefore, diverse and functionally overlapping sets of genes are altered in asthmatics, though their precise roles in pathogenesis remain to be defined.

### iRegulon

Up-regulated genes in the asthmatic group were highly enriched in E2F-linked transcription factor-binding motifs: *E2F8* was most significantly up-regulated, followed by *E2F2* and *E2F3*. Members of the E2F transcription factor family are key players in cell cycle regulation^36^. E2F8 is thought to be a transcriptional repressor of DNA-damage responses in cancer^37^, and also to have opposing roles as oncogene or tumor suppressor under specific conditions^38^. E2F2 and E2F3 are transcriptional activators^36^. Nuclear overexpression of E2F3 has been associated with lung cancer^39^, and E2F2 was identified in non-small cell lung carcinoma in humans^40^. In non-asthmatic horses the transcriptional activator *E2F1* was significantly down-regulated^28^. FOXM1 in part activates CDC25A through E2F transcription factor-pathways^31^. Interestingly, in humans, E2F1 inhibition was linked to reduced airway SMC proliferation^41^. Hence, data presented here suggest that E2F8, E2F2 and E2F3 regulate cell proliferation in epithelial cells of asthmatic horses.

Transcription factor-binding motifs containing *RFX* were overrepresented among significantly down-regulated genes in both groups. Specifically, *RFX2* was down-regulated in both groups while *RFX5* was down-regulated in asthmatics only. RFX2 is involved in ciliogenesis^42^, while RFX5 regulates expression of MHC II genes^43,44^. These findings suggest that MHC II may have a limited role in the exacerbation phase of asthma and that ciliary regeneration may be impaired in equine asthma, as also described in human asthma^45^.

### Equine versus human severe asthma

Clinical features of severe equine asthma closely resemble those of low-Th2 late-onset human asthma. The pathogenesis of this particular human asthma phenotype remains poorly understood, which precludes devising effective interventions. In this study of equine asthma, gene sets including but not limited to neutrophil migration and chemotaxis, Hh signalling, wound healing, hemostasis, coagulation and regulation of body fluid, were significantly enriched in the asthmatic group following challenge. Enrichment of neutrophil migration and chemotaxis gene sets is consistent with influx of neutrophils into airway lumens during exacerbation, and with differential expression of IL8 and CXCR2 in asthmatic and non-asthmatic horses^26^. Neutrophils are the prevailing luminal leukocyte in equine asthma, and also predominate in most late-onset human asthma^9,46^. Yick *et al*., using RNA-seq of endobronchial biopsies from human asthmatics and controls, found comparable differential expression of genes in networks concerning cellular movement, cell death and cellular morphology, genetic disorder, and cellular movement and development^47^.

Following epithelial injury, inflammation and tissue repair mechanisms in the bronchial epithelium are activated. Resolution of inflammation is crucial for orderly epithelial tissue repair^48^, but over-expression of innate immune response, tissue repair, cell cycle, Hh signalling and other genes intimates that re-establishment of epithelial homeostasis fails in exacerbated asthma. Hence, we propose that chronic inflammation causes dysregulation of the epithelial cell cycle and of progenitor cell differentiation, which in turn promotes tissue remodeling and SMC proliferation characteristic of advanced asthma. Hh signalling modulates cell cycle and remodelling processes through expression of PTCH1^49^. While the Hh pathway is crucial for compartmentalization in the developing lung, additional roles in adult lung diseases were recently identified. SNPs close to the negative regulator hedgehog interacting protein (*HHIP*) on 4q31 were associated with decreased lung function and lower HHIP promoter activity in COPD patients^50^. In humans with chronic lung fibrosis, Hh was expressed specifically at affected epithelial sites, and the Hh receptor PTCH1 on infiltrating leukocytes^51^. SNPs in *PTCH1* and *HHIP* regions were associated with decreased lung function in asthmatic patients^52^. Together, these results support the concept that impaired Hh signalling in horses and humans may be a causal factor in pathologic airway remodelling through dysregulation of cell cycle and differentiation. However, the Hh pathway is unlikely activated in isolation, and the Wnt pathway is a possible candidate also linked to asthma susceptibility. In a genome-wide association study (GWAS) of asthmatic humans the Wnt signalling pathway was significantly enriched, and multiple SNPs near Wnt interacting genes were strongly associated with asthma susceptibility^53^. The Wnt/catenin pathway has been considered to regulate fibrosis and SMC remodelling in a mouse model of asthma^54^. In this study, two Wnt signalling gene sets were significantly enriched in asthmatic horses following challenge. Since Wnt and Hh signalling pathways co-exist and interact in diseases such as colon cancer, it is conceivable that both may contribute to aberrant epithelial repair and concomitant stromal cell abnormalities in asthma^55^.

Finally, based on target enrichment analysis, we suggest that E2F transcription regulates gene expression in the asthmatic lung. E2F2, E2F3 and E2F8 were significantly up-regulated in asthmatic horses only, while E2F1 was significantly down-regulated in non-asthmatics. E2F transcription factors are crucial in regulating cell cycle^56^, lung development^57^ and transcriptional programs for inflammation, immunity, metabolism and stress-response^58^. E2Fs factors can have redundancy in function through either promotion or repression of transcription, sometimes shifting function under different conditions, such as different development stages^59^. For instance, E2F8, a known transcriptional repressor^60,61^ can suppress E2F1^62^ but is overexpressed in lung cancer^63^. Overall, E2F transcription is complex and particular transcription factors have not yet been directly linked to human or equine asthma. Chromatin immunoprecipitation and protein-protein interaction assays with specific E2Fs antibodies could more specifically clarify their roles in asthma.

## Conclusion

We assessed changes in gene expression in the bronchial epithelium within groups of asthmatic and non-asthmatic horses after a dusty hay challenge. In asthmatic horses, we observed enrichment of 587 gene sets and transcription factor targets linked to activation of cell cycle and inflammation/immune response programs. A small number of cell cycle gene sets were also significantly enriched in non-asthmatics, but restricted to M phase of the cell cycle. These findings point at mechanisms underlying impaired regeneration of the epithelial barrier in asthmatics, and may be pivotal events leading to subepithelial tissue remodeling and eventual irreversible lung parenchymal changes. Furthermore, gene sets and transcription factor targets affecting cilia were significantly enriched among down-regulated genes in both groups. Although interesting, the biological significance of this findings is unknown and awaits further investigation.

Additional time-points would have yielded a more dynamic representation of tissue response, and might have more precisely defined the divergence of physiological from pathological epithelial changes. Such data might also allow analysis of dynamic changes in specific pathways. Tissue factor motifs identified as enriched were consistent with gene set changes, but the biological role of their cognate binding partners needs to be confirmed with functional assays.

## Methods

### Animals and procedures

Overall study design is outlined in Fig. 1. Animal procedures, sample collection and sample processing in this study were performed as previously described by Tessier *et al*.^28^. Briefly, six horses with asthma in remission and seven horses without asthma (mean ages of 15 and 12 years (*p* = 0.352, unpaired t test, respectively) were placed in a dust-free indoor environment for 24 hours before exposure to dusty hay until respiratory impairment was apparent in asthmatic horses (range 1 to 3 days, average 2.2 days). Non-asthmatic horses were exposed to dusty hay for 3 days. Physical examination, pulmonary function test (PFT) and bronchoalveolar lavage (BAL) were performed before and after exposure to the asthmatic challenge, and endoscopic bronchial biopsies were obtained from a contralateral lung lobe. All procedures were approved by the Institutional Animal Care Committee of the University of Guelph (protocol R10-031) and conducted in compliance with Canadian Council on Animal Care guidelines.

### RNA-Seq sample preparation and analysis

RNA extraction, RNA-seq library generation and sequencing were performed as previously described^28^. Briefly, total RNA was extracted from endobronchial biopsies (Qiagen, Toronto, ON). RNA quality and concentration was determined with the Bioanalyzer RNA Nanochip (Agilent, ON) and capillary electrophoresis. RNA-seq unstranded library preparation and sequencing were performed at The Centre for Applied Genomics (TCAG; Toronto, ON) using the Illumina TruSeq RNA sample preparation and sequencing protocols (Illumina, San Diego, CA). For each sample, approximately 1 μg of non-degraded, high quality total RNA was enriched for poly-A RNA, fragmented into 200 to 300 bases, and converted to double stranded cDNA libraries. Final RNA libraries were quantified (KAPA Library Quantification kit, Kapa Biosystems, Wilmington, MA) prior to pooling and sequencing. Illumina flow cells were prepared and samples sequenced on an Illumina HiSeq 2500 instrument in 5 lanes following the manufacturer’s instructions to generate paired-end reads of 100-bases.

Raw read quality was assessed using FastQC software version 0.10.1 (http://www.bioinformatics.babraham.ac.uk/projects/fastqc/) and aligned to the horse reference genome^64^ (Ensembl v70) with STAR version 2.4^65^. The STAR_pass2 alignment protocol was followed including these adaptations: horse Ensembl version 70 GTF annotation file for first- and second-pass, and the junction SJ.tab file generated by STAR for the second-pass after non-canonical junctions were removed. Default settings were used except for:–runThreadN 8–outFilterScoreMinOverLread 0.5–outFilterMatchNminOverLread 0.5. Read counts were generated from STAR alignment files using HTSeq version 0.6.1p1^66^ with settings -s no -f bam -r name.

### Statistical Analysis

Differential expression (DE) analysis was performed in R, version 3.2.1 (www.r-project.org), with the edgeR package version 3.10.2^67–69^ as previously described^28^ with exception of the applied contrast. Paired DE analysis was performed to assess changes within groups (before versus after asthmatic challenge in asthmatics and non-asthmatics separately). The edgeR analysis was based on section 3.5 of the edgeR user’s guide (last revised April 10, 2017). The model matrix was designed as followed: ~group + group:horse + group:challenge, where “group” refers to non-asthmatic and asthmatic groups, “horse” refers to each horse, and “challenge” refers to samples before and after the asthmatic challenge. Fit of the generalized linear model and tests for differences in expression were performed with the “glmFit” and “glmLRT” functions, respectively. For the current study, GlmLRT(fit, coef = “groupNonAsthmatic:postChallenge”), and glmLRT(fit,coef = “groupasthmatic:postChallenge”) were used to analyze asthmatic challenge effect within the non-asthmatic and asthmatic horse group (before challenge versus after challenge), respectively. Statistical significance was set at a false discovery rate (FDR) < 0.05.

### Gene set enrichment, modules and network analysis

Ranked Gene Set Enrichment Analysis (GSEA) was performed with software version 2.1.1^25,70^. The human gene set file excluded annotations with evidence codes - ‘IEA’ (inferred from electronic annotation), ‘ND’ (no biological data available), ‘RCA’ (inferred from reviewed computational analysis) and was downloaded from: http://download.baderlab.org/EM_Genesets/January_28_2015/Human/symbol/Human_GO_AllPathways_no_GO_iea_January_28_2015_symbol.gmt^71^.

GSEA pre-ranked analysis (GseaPreranked) was performed using default settings except for “Collapse dataset to gene symbols” set to “False”. Prior to analysis, a ranked list was calculated with each gene assigned a score based on the FDR and the direction of the log fold-change (“+” or “−”). Horse Ensembl IDs were converted to HUGO gene symbols. Non-matching symbols were enriched using the human orthologs when percent identity was above 80% for target and query sequence. Input gene and score lists for asthmatic and non-asthmatic horses are included in Suppl. Files 1 and 2, respectively. Gene sets identified as significant (FDR < 0.05, *p* < 0.001) with GSEA were visualized using the Enrichment Map plugin available for Cytoscape version 3.4.0^26,72^. Connected nodes only were included, and gene set clusters were summarized and labeled manually with the WordCloud plugin^73^ for Cytoscape version 3.4.0^72^.

### Transcription factor target enrichment

Transcription factor target enrichment among differentially expressed genes in asthmatic and non-asthmatic horses was performed with Cytoscape (v 3.4.0) in combination with the iRegulon plugin^74^. Analysis was conducted using the *Homo sapiens* database and default settings. Genes with FDR < 0.05 were included in the analysis, and up- and down-regulated genes were analyzed separately. iRegulon input for each of the groups is included in Suppl. Files 3–6. Transcription factors of interest with motif enrichment scores (NES) > 3 were selected for further analysis.

The datasets generated and analyzed in this study are available in the NCBI Sequence Read Archive at https://www.ncbi.nlm.nih.gov/Traces/study/?acc=SRP106023.

## Electronic supplementary material

Supplementary Information
